# A Role for Glutamate Transporters in the Regulation of Insulin Secretion

**DOI:** 10.1371/journal.pone.0022960

**Published:** 2011-08-11

**Authors:** Runhild Gammelsaeter, Thierry Coppola, Païkan Marcaggi, Jon Storm-Mathisen, Farrukh A. Chaudhry, David Attwell, Romano Regazzi, Vidar Gundersen

**Affiliations:** 1 Department of Anatomy and the CMBN, University of Oslo, Oslo, Norway; 2 Institut de Pharmacologie Moléculaire et Cellulaire, Université de Nice, Nice, France; 3 Department of Physiology, University College London, London, United Kingdom; 4 Département de Biologie Cellulaire et de Morphologie, Université de Lausanne, Lausanne, Switzerland; 5 The Biotechnology Centre of Oslo, University of Oslo, Oslo, Norway; 6 Department of Neurology, Rikshospitalet, Oslo, Norway; University of Bremen, Germany

## Abstract

In the brain, glutamate is an extracellular transmitter that mediates cell-to-cell communication. Prior to synaptic release it is pumped into vesicles by vesicular glutamate transporters (VGLUTs). To inactivate glutamate receptor responses after release, glutamate is taken up into glial cells or neurons by excitatory amino acid transporters (EAATs). In the pancreatic islets of Langerhans, glutamate is proposed to act as an intracellular messenger, regulating insulin secretion from β-cells, but the mechanisms involved are unknown. By immunogold cytochemistry we show that insulin containing secretory granules express VGLUT3. Despite the fact that they have a VGLUT, the levels of glutamate in these granules are low, indicating the presence of a protein that can transport glutamate out of the granules. Surprisingly, in β-cells the glutamate transporter EAAT2 is located, not in the plasma membrane as it is in brain cells, but exclusively in insulin-containing secretory granules, together with VGLUT3. In EAAT2 knock out mice, the content of glutamate in secretory granules is higher than in wild type mice. These data imply a glutamate cycle in which glutamate is carried into the granules by VGLUT3 and carried out by EAAT2. Perturbing this cycle by knocking down EAAT2 expression with a small interfering RNA, or by over-expressing EAAT2 or a VGLUT in insulin granules, significantly reduced the rate of granule exocytosis. Simulations of granule energetics suggest that VGLUT3 and EAAT2 may regulate the pH and membrane potential of the granules and thereby regulate insulin secretion. These data suggest that insulin secretion from β-cells is modulated by the flux of glutamate through the secretory granules.

## Introduction

Glutamate is the transmitter used at most excitatory synapses in the brain [1]. Glutamate is carried from the cytosol into synaptic vesicles by vesicular glutamate transporters (VGLUTs) in a process dependent on a vacuolar ATP-driven H^+^ pump. There are three subtypes of VGLUT [2]. In the brain VGLUT1 and VGLUT2 are confined to known glutamatergic pathways, whereas VGLUT3 is also present in non-glutamatergic neurons [3]. Transport of glutamate (and aspartate) from the extracellular fluid into the cytosol of brain cells is accomplished by a family of sodium dependent excitatory amino acid transporters (EAATs 1–5), which are found in plasma membranes [4], [5].

The role of glutamate transmission in non-neuronal organs has been less investigated. In the pancreatic islets of Langerhans, which consist mainly of insulin secreting β-cells and glucagon secreting α-cells, several components of a glutamate signalling system have been identified [6]–[8]. In α-cells VGLUT1 and VGLUT2 are reported to package glutamate into secretory granules prior to exocytotic release together with glucagon [8]. In β-cells, however, a novel role of glutamate has been proposed. Glutamate acting through a VGLUT is thought to regulate insulin secretion [9], [10] without itself being secreted [8]. The molecular identity and ultrastructural localization of the vesicular transporter, as well as the concentration of glutamate in β-cell secretory granules, are unknown. Expression of mRNA for EAAT2 has also been reported [11], but the location of these transporter proteins in islet cells is unresolved.

To study the role of glutamate transport processes in insulin secretion, we used high resolution immunocytochemistry and EAAT2 knock out (KO) mice, in combination with functional secretion studies exploiting small interfering RNA (siRNA) techniques and simulations of granule energetics. Our data suggest a glutamate cycle in which glutamate is carried into the granules by VGLUT3 and carried out by EAAT2. The concerted action of these glutamate transporters may regulate the pH and membrane potential of the granules and thereby modulate insulin secretion.

## Results and Discussion

### VGLUT3 is present in membranes of insulin containing secretory granules

**Figure 1:**
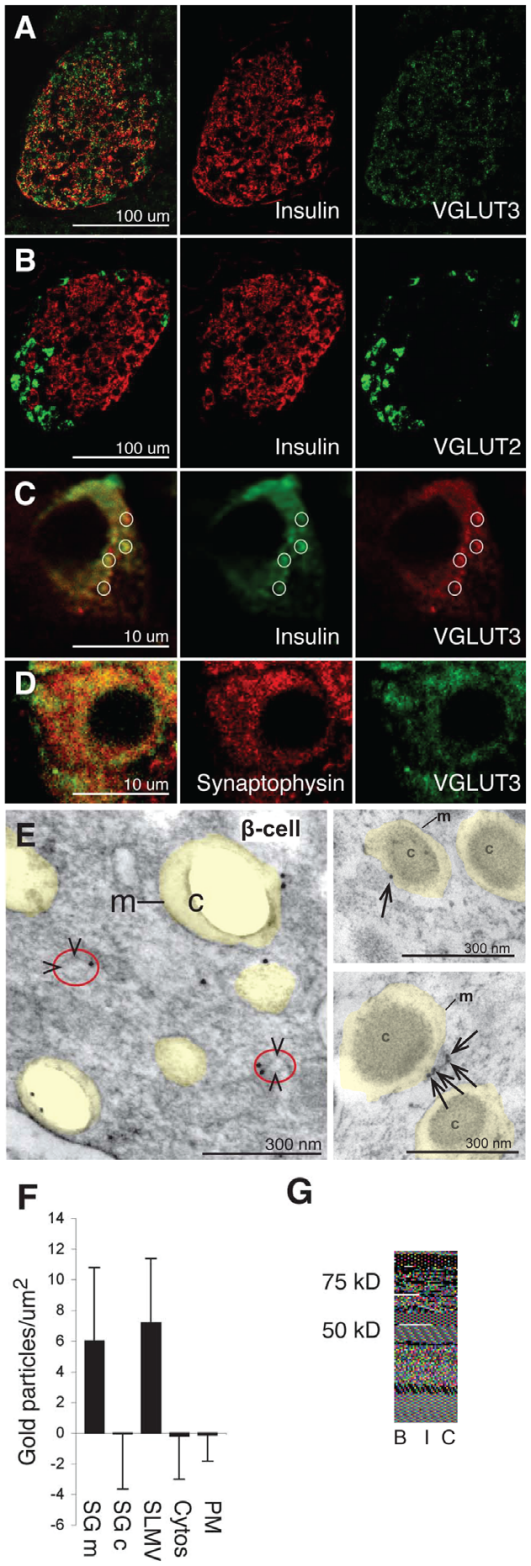
The vesicular glutamate transporter VGLUT3 is localized in secretory granules and synaptic-like microvesicles (SLMVs) in pancreatic β-cells. (A) VGLUT3 (green) co-localizes with insulin (red), but is also found in peripheral non-B islet cells. (B) VGLUT2 (green) does not co-localize with insulin (red). (C) A single β-cell from the islet presented in panel A: VGLUT3 (red) co-localizes partly with insulin (green). The circles highlight some of the overlapping VGLUT3 and insulin dots. (D) A single β-cell: VGLUT3 (green) co-localizes partly with synaptophysin (red) in small dots. (E) Electron micrograph showing immunogold particles for VGLUT3 in two β-cells. Secretory granules are indicated by transparent yellow. m, membranes of secretory granules. c, core of the secretory granule (of which some disappeared in the preparation procedure). SLMVs are indicated by arrowheads and red circles. (F) Quantification of VGLUT3 in β-cells (n = 8 cells). Immunogold particle densities (mean number of gold particles/µm^2^ ±SD) in the membrane of the granules (SGm) and in SLMVs are significantly higher than in the core of the β-cell granules (SGc), cytosol and plasma membranes (PM) (background labeling, quantified over mitochondria, is subtracted, see Methods) (p<0.001, Mann-Whitney-U test, two tails). (G) Western blots of rat brain tissue (B) and isolated rat islets (I) probing for VGLUT3. (C), islet blot without the VGLUT3 primary antibodies.

Immunoblots revealed that VGLUT3 is present in islet tissue (Figure 1G), and immunofluorescent microscopy demonstrated that VGLUT3 is present in both β-cells and α-cells (Figure 1A), whereas VGLUT2 is present in α-cells only (Figure 1B). We could not detect VGLUT1 in any islet cell type (not shown). Thus, the only VGLUT detected in the β -cells is VGLUT3. VGLUT3 was co-localized with insulin containing granules (Figure 1A, C), and there was also a significant co-localization with the synaptic-like microvesicle (SLMV) marker synaptophysin (Figure 1D). Electron microscopic immunogold labelling confirmed that VGLUT3 is located in both secretory granules and SLMVs (Figure 1E). Quantitative immunogold analysis showed that the VGLUT3 labelling densities in the membranes of secretory granules and SLMVs were much higher than in plasma membranes or in cytosol (where the densities were not significantly different from background labelling over mitochondria: Figure 1F). Consistent with these immunocytochemical data, subcellular fractionation of cells from the β -cell insulinoma line INS-1E showed that VGLUT3 is present in secretory granules as well as in SLMVs (Figure S1). These data suggest that VGLUT3 is the previously hypothesized [9], [10] protein that mediates glutamate uptake into insulin containing secretory vesicles.

### The glutamate levels in insulin granules are low

**Figure 2:**
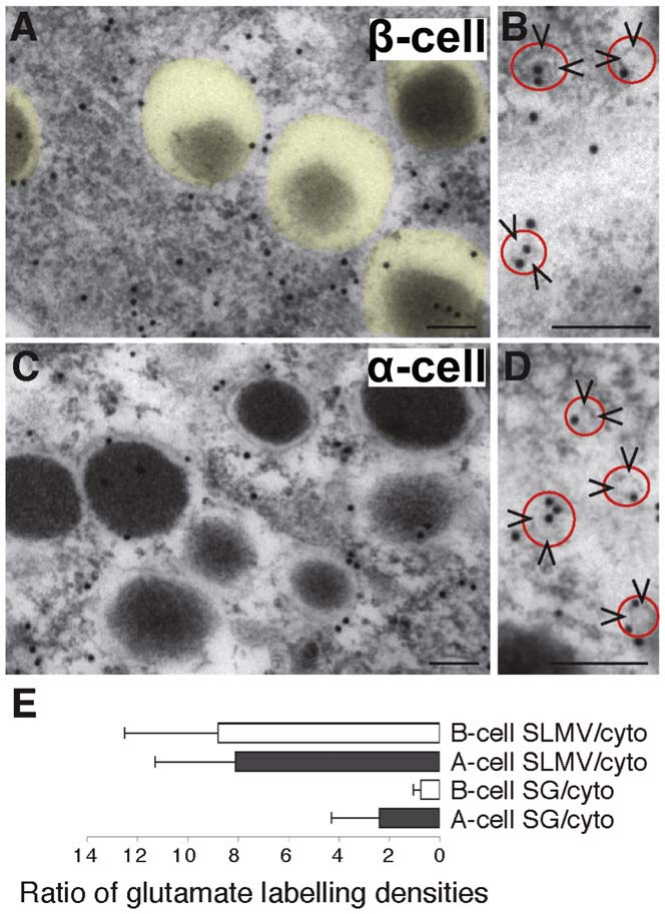
The glutamate concentration in SLMVs is much higher than in secretory granules, and lower in β-cell secretory granules than in α-cell secretory granules. (A) Immunogold particles representing glutamate in β-cell cytoplasm are scarce over secretory granules (transparent yellow). (B) Close up showing glutamate gold particles in β-cell SLMVs (arrowheads, red circle). (C) Immunogold particles representing glutamate in α-cell cytoplasm. (D) Close up showing glutamate gold particles in α-cell SLMVs (arrowheads, red circle). Scale bars A–D, 100 nm. (E) Immunogold quantification shows that the secretory granule (SG)/cytosol (cyto) ratio (mean±SD) of net glutamate labelling (background subtracted, see Methods) is significantly lower in β-cells than in α-cells (p<0.05, n = 5 cells of each kind) and that the SLMV/cytosol ratio is much higher than the SG/cytosol ratio in both α- and β-cells (p<0.01, n = 5 cells of each kind) (Mann-Whitney-U test, two tails). The mean glutamate labelling density (average number of gold particles/µm^2^±SD) in α-cell granules was 33.6±14.9, whereas the value in α-cell cytosol was 21.1±4.6 (p<0.05, Mann-Whitney-U test, two tails). In β-cells the glutamate densities were 19.7±12.4 in the granules and 30.2±7.6 in the cytosol (p<0.05, Mann-Whitney-U test, two tails). From ultrathin test sections with conjugates containing known concentrations of glutamate, which were processed with the glutamate antibodies in parallel with the glutamate labelling of islet tissue, a relationship between the concentration of fixed glutamate and the gold particle density in islet tissue can be approximately estimated. In β-cells the approximate concentration of glutamate was estimated to be in the lower mM range (2–3 mM in secretory granules and cytosolic matrix, respectively).

If VGLUT3 transports glutamate into secretory granules, we would expect a high concentation of glutamate in the granules. Surprisingly, although electron immunogold microscopy using antibodies to glutamate showed that glutamate is indeed located in secretory granules in β-cells, it is present at lower levels than in the cytosol and much lower than in SLMVs (Figure 2A–B, E). In α-cells the glutamate concentration is also high in SLMVs (Figure 2C–D), but in contrast to the situation in β-cells, the glutamate levels in the granules are higher than in the cytosol (Figure 2E). In B cells the granule∶cytosol ratio for glutamate is about 0.70, compared to about 2.4 in α-cells (Figure 2E, significantly different, p<0.05). Both ratios are significantly different from 1 (Mann Whitney U test, p<0.05), suggesting that the glutamate levels in the α- and β-granules are higher and lower, respectively, than in the cytosol.

### EAAT2 is present in insulin granules

**Figure 3:**
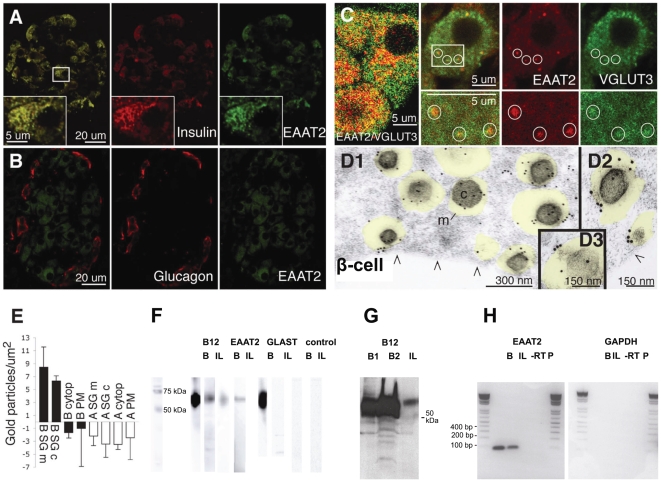
The glutamate transporter EAAT2 is selectively localized in β-cell secretory granules. (A–B) EAAT2 (green) co-localizes with insulin (red in A) in β-cells but not with glucagon in α-cells (red B). (C) EAAT2 (red) co-localizes partly with the vesicular glutamate transporter VGLUT3 (green). At higher magnification (right panels) it is evident that there are granules containing both EAAT2 and VGLUT3, some of which are indicated by circles. (D1–D3) Electron micrographs showing that EAAT2 immunogold particles are localized in secretory granules (transparent yellow) in three different β-cells. m, granule membrane. c, granule core. Arrowheads (/\), plasma membrane. E, Quantification of EAAT2 in different cellular compartments in α- and β-cells. The values are mean number of EAAT2 gold particles/µm^2^±SD in the various tissue compartments in 7 α- and 7 β-cells. The EAAT2 density is significantly higher in the membrane (B SG m) and core (B SG c) of the granules in β-cells, than in the cytosol of α- and β-cells (A cyto and B cyto), the plasma membrane of α- and β-cells (A PM and B PM) and the core (A SG c) and limiting membrane (A SG m) of α-cell granules (p<0.001, Mann-Whitney-U test, two tails). (Background labelling was subtracted, see Methods. Except for B SG m and B SG c, all other compartments observed were at background levels.) The quantitative data presented are from one animal and similar results were obtained in two other animals. F, Western blots of isolated rat pancreatic islets (I) and rat brain tissue (B) using EAAT2 antibodies raised against both the C-terminal (B12) and the N-terminal (monoclonal (M)) parts. EAAT1 showed no band in islet tissue. Controls (C) without primary antibody showed no band. Note that the Westerns were run with several different homogenate concentrations, so that the lanes to be directly compared could mostly not be on the same membrane, however all membranes were run in the same set of experiments. (G) Western blot of isolated rat pancreatic islets (I), and rat brain tissue (2 separate brains, B_1_ and B_2_) all on the same membrane using EAAT2 antibodies raised against the EAAT2 C-terminal (B12). (H) RT-PCR of rat brain tissue (B) and isolated islets (I) in which there was a PCR signal for EAAT2 at about 100 bp (expected value), but not for human GAPDH.

**Figure 4:**
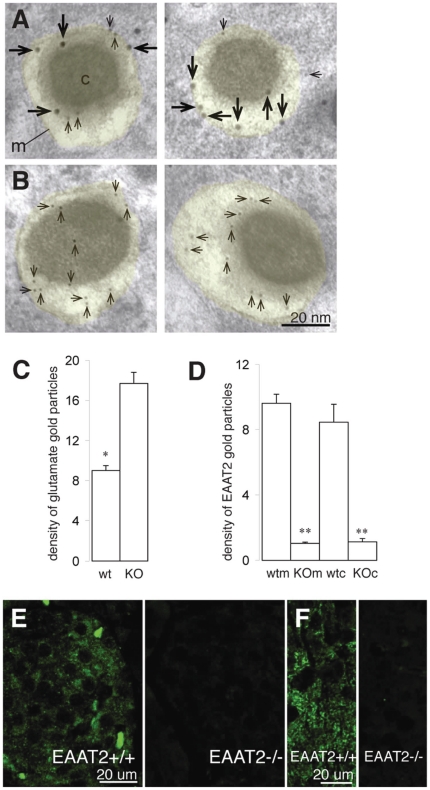
Lack of EAAT2 and increased concentration of glutamate in secretory granules in EAAT2 KO mice. (A–B) Immunogold electron micrographs showing EAAT2 (large gold particles, long arrows) and glutamate (small gold particles, short arrows) in β-cell secretory granules (transparent yellow) in wt (A) and KO (B) islets (two examples of secretory granules shown for each phenotype). m, granule membrane. c, granule core. (C–D) Quantitative representation of the glutamate (C) and EAAT2 (D) gold particle densities in β-cell secretory granules in wild type (wt) and knock out (KO) mice. The values are mean numbers of gold particles/µm^2^±SEM in 4 KO and 4 wt animals (background subtracted, see Methods). 119 secretory granules in wt mice and 179 secretory granules in KO mice were included in the quantifications. *, the value in KO secretory granules is significantly higher than the value in wt secretory granules (p<0.01, Mann-Whitney-U test, two tails) and **, the values in the limiting membrane (m) and the core (c) of the secretory granules are significantly lower in KO than in wt mice (p<0.01, Mann-Whitney-U test, two tails). (E–F) Islet (E) and brain (F) tissue from wild type (EAAT2+/+) and EAAT2 KO (EAAT2−/−) mice labelled with the EAAT2 antibodies.

The observation that the glutamate concentration is low in β-cell secretory granules, despite the presence of VGLUT3, motivated a search for proteins that could actively transport glutamate out of the secretory granules. The plasma membrane glutamate transporters could be such proteins. We performed immunocytochemical experiments with antibodies against the plasma membrane glutamate transporters known to be present in the brain or in non-neuronal tissues (EAAT1, EAAT2, EAAT3 and EAAT4); as EAAT5 is restricted to the retina [12], this transporter was not included in the study. Of these transporters only EAAT2 was detected in the endocrine pancreas. EAAT2 is present only in insulin positive β-cells (Figure 3A) and not in glucagon positive α-cells (Figure 3B). In contrast to what is observed in the brain [4], EAAT2 was not present at the cell membrane, but co-localized with insulin in secretory granule-like dots in the cytoplasm (Fig. 3A inset). This staining pattern was found with all available EAAT2 antibodies (the anti-B12, anti-B483, anti-B518, anti-B568, and anti-73kDa antibodies, as well as the monoclonal 9C4 antibody, which are described in the Methods), directed to different parts of the protein and in several species (rat (Fig. 3A, B, C), mouse (Fig. 4E) and human (not shown)).

In order to directly visualize and quantify EAAT2 in insulin containing granules in β-cells we performed electron microscopic immunogold cytochemistry. A high density of EAAT2 gold particles was detected in β-cell secretory granules (Figure 3D, E), while α-cell secretory granules showed a labelling density at background level (Figure 3E). The EAAT2 level was high both in the granule limiting membranes and in the cores of the secretory granules (perhaps suggesting that there is some sort of protein reservoir within the granules), but very low both in α- and β-cell plasma membranes and in the cytosol of both cell types (Figure 3D, E). Furthermore, EAAT2 was found to be co-localized with VGLUT3 (Figure 3C), suggesting that both types of glutamate transporter are situated in the same secretory granule.

The presence of EAAT2 protein in the islet tissue was detected with antibodies against different epitopes of EAAT2 on Western blots from acutely isolated islets, giving bands with an appropriate molecular mass [13] (Figure 3F, 3G), and at the mRNA level using RT-PCR (Fig. 3H). In contrast, EAAT1 (Figure 3F) and EAAT3 (not shown) immunoblots were negative. An additional demonstration of the presence of EAAT2 in β-cell secretory granules was obtained from Western blots of subcellular fractions from INS-1E cells, which showed that the EAAT2 antibodies only labelled the insulin-containing fractions and not the synaptophysin-containing ones (Figure S1).

### EAAT2 keeps the glutamate levels in the insulin granules low

Based on the data above, we propose that there is a flux of glutamate through the secretory granules provided by VGLUT3, which mediates uptake of glutamate into the granules, and EAAT2, which carries glutamate out of the granules back into the cytosol. To further test this hypothesis we labelled ultrathin sections from EAAT2 knock out (KO) and wild type (wt) [14] mice with antibodies against EAAT2 and glutamate using the immunogold method (Figure 4A–D). In the KOs the EAAT2 signal in β-cell secretory granules was at background level (Fig. 4B, D), while the glutamate levels were twice as high as in the wt mice (Figure 4A–C), consistent with an abolition of glutamate export from the granules. The level of glutamate in β-cells was significantly lower in the granules than in the cytosol in the wt mice (9.0±0.9 vs. 12.2±2.1 gold particles/µm^2^±SEM p<0.05, n = 4 mice), confirming the glutamate data from the rats (Figure 2E), whereas the opposite was observed for the KO mice (17.7±2.0 vs. 10.5±3.9 gold particles/µm^2^±SEM, p<0.05, n = 4 mice). The wt mice showed an EAAT2 labelling pattern similar to that in the rats (compare Figures 4E and 3A).

To further confirm the EAAT2 results, we immunofluorescently labelled islets and brains from KO and wt mice. Islets from wt mice show EAAT2 labelling in β-cells, whereas there was no significant EAAT2 labelling in islets from KO mice (Figure 4E). Brain sections, processed with the EAAT2 antibodies along with the islet sections, showed labelling of the wt brain and no labelling of the KO brain (Figure 4F) [15], further substantiating that the EAAT2 antibodies give a specific labelling signal in the tissue.

### EAAT2 carries glutamate out of the granules

**Figure 5:**
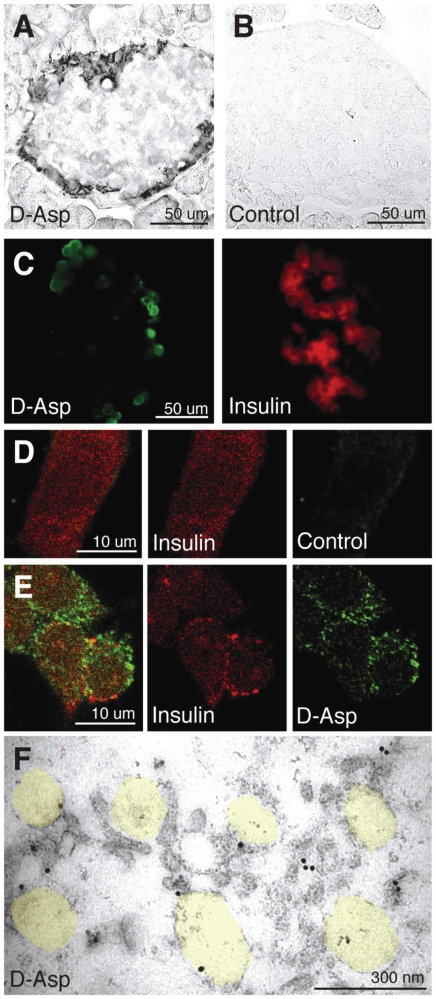
The glutamate analogue D-aspartate is neither taken up through the plasma membrane in intact β-cells nor into SGs in permeabilized β-cells. A–C, Acutely prepared slices of islet tissue were incubated with exogenous D-aspartate (100 µM) before aldehyde fixation and labelling with antibodies that selectively recognize D-aspartate. (A–B) Immunoperoxidase labelling shows that the tissue not exposed to D-aspartate (Control) is unlabelled, while in islets exposed to D-aspartate (D-Asp) labelling is observed only in the peripheral α-cell area, not in the central β-cell area of the islet. (C) Immunofluorescence shows that the central insulin positive β-cells are negative for exogenous D-aspartate and that the peripheral non-insulin α-cells are labelled. (D–F) Streptolysin-*O* permeabilized INS-1E cells were exposed to different concentrations of exogenous D-aspartate (0–3 mM) before fixation and labelling with the D-aspartate antibodies. (D) In cells not exposed to D-aspartate (Control) there was no labelling for D-aspartate, only for insulin (red). (E) In cells exposed to 1 mM D-aspartate staining with the D-aspartate antibodies (green) is observed. There was some weak co-localization (yellow) with insulin (red) that is attributable to extra granular fixation of D-aspartate (see F). (F) Electron micrograph of permeabilized INS cells exposed to 1 mM D-aspartate shows no significant D-aspartate labelling inside the secretory garnules (indicated in transparent yellow). Note some labelling along the limiting membrane of secretory granules and in the cytosol, reflecting fixation of exogenous D-aspartate to extragranular proteins.

As we could not detect any immunocytochemical signal for EAAT2 (or any of the other known glutamate transporters) in the β-cell plasma membrane, we tested for glutamate transport activity in the plasma membrane using exogenous D-aspartate as a tracer [16], since this is transported by all the EAATs. We could not find any evidence for transport across the β-cell plasma membrane (Figure 5A–C), supporting the idea that EAAT2 has a function other than plasma membrane transport in β-cells. The lower glutamate content in the β-cell secretory granules than in the cytosol (Figure 2E), and the increase in granule glutamate level when EAAT2 is knocked out (Figure 4C), suggest that EAAT2 is not carrying glutamate from the cytosol into the secretory granules, but in the opposite direction. To test for transport from the cytosol into the granule, we employed cells of an insulin secreting cell line (INS-1E cells) that, like intact β-cells, express EAAT2 (not shown). These cells were permeabilized with streptolysin-*O* to allow entry into the cytosol of exogenous D-aspartate (which is a substrate for EAAT2 [4], [5], [16], but not for the VGLUTs, including VGLUT3 [3], [17], [18]). Immunofluorescence and immunogold staining showed that exogenous D-aspartate was not co-localised with insulin, but rather represents D-aspartate fixed to extragranular compartments (Figure 5E and 5F).

Thus, we conclude that EAAT2 is not transporting glutamate into the secretory granules, but instead mediates extrusion of glutamate from the vesicular lumen to cytosol. Glutamate transport by EAAT2 is coupled [19] to the co-transport of 3 Na^+^ and 1 H^+^ and the counter-transport of 1 K^+^. Secretory granules contain high concentrations of H^+^ compared with the cytosol which, along with a positive granule membrane potential relative to the cytoplasm generated by the vacuolar H^+^-ATPase, will favour transport of glutamate out of the granule by EAAT2. Little is known about the intragranular concentration of sodium in insulin containing secretory granules, however secretory granules in neurohypophysial cells have been shown to contain sodium [20]. Thus, the membrane potential and ion concentration gradients across the membranes of insulin-containing secretory granules may be in favour of an outward (lumen-to-cytosol) transport of glutamate.

### EAAT2 and VGLUT regulate insulin secretion

**Figure 6:**
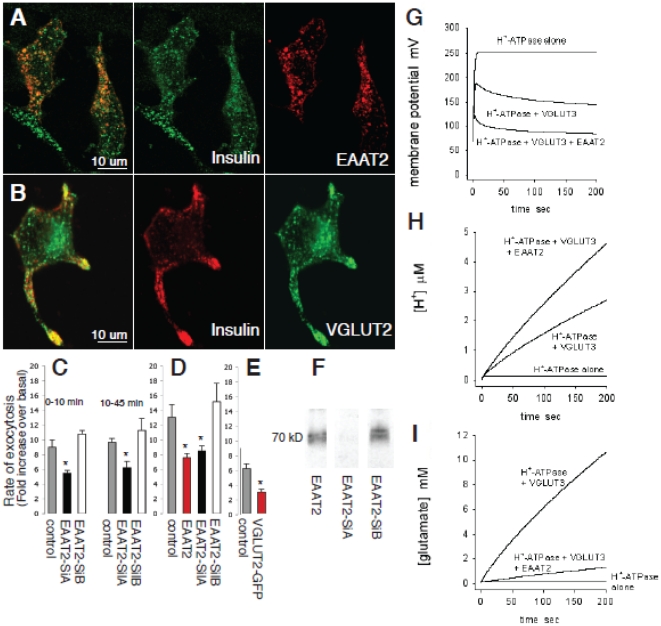
The effect of EAAT2 and VGLUT expression on secretory granule energetics and exocytosis. (A) INS-1E cells were transfected with EAAT2-c-Myc and the EAAT2 protein was visualized with anti-c-Myc antibodies (red). EAAT2 co-localized with insulin (green). (B) INS-1E cells were transfected with GFP-VGLUT2 and VGLUT2 visualized by the fluorescence of the GFP tag. VGLUT2 (green) co-localized with insulin (red). (C) INS-1E cells were transiently co-transfected with a plasmid encoding hGH and with vectors directing the sequence of two different short interfering RNAs for EAAT2 (EAAT2 SiA and EAAT2 SiB) or with empty pSUPER vector (control). The cells were incubated under basal and stimulatory conditions (see methods) for 10 and 45 min. hGH secretion was measured by ELISA as the fold increase over basal conditions. The EAAT2 SiA values were significantly different from control and EAAT2 SiB values (*, unpaired Student's t-test, p<0.05, n = 3). (D) In another set of experiments INS-1E cells were cotransfected with hGH and the pSUPER control vector alone (control) or with EAAT2, EAAT2 SiA or EAAT2 SiB, and incubated for 45 min as in C. EAAT2 and EAAT2 SiA values were significantly different from control and EAAT2 SiB values (*, unpaired Student's t-test, p<0.05, n = 3). (E) INS-1E cells were transiently co-transfected with hGH and VGLUT2-GFP or GFP (control). The VGLUT2 value was significantly different from the control value (*, unpaired Student's t-test, p<0.05, n = 3). (F) INS-1E cells were cotransfected with c-Myc-tagged EAAT2 and the two siRNAs targeting different sequences of EAAT2 (EAAT2 SiA and EAAT2 SiB). The Western blots show that the EAAT2 SiA almost completely blocked the expression of EAAT2, whereas EAAT2 SiB had no effect. (G–I) Simulations of the effect of VGLUT3 and EAAT2 on granule energetics. Granule cell membrane potential (G), [H^+^] (H) and [glutamate] (I) are shown for a granule membrane containing the H^+^-ATPase alone, ATPase and VGLUT3, or ATPase, VGLUT3 and EAAT2 (see supplementary text S1).

To assess the function of EAAT2 in the regulation of insulin secretion from β-cells, we diminished its expression level in INS-1E cells using RNA interference [21], [22]. We generated two plasmids that allow the synthesis of short double-stranded RNA molecules (siRNAs) directed against different sequences of rat EAAT2. As shown in Figure 6F, transient transfection of EAAT2-SilA led to a very strong reduction of rat EAAT2 expression. In contrast, EAAT2-SilB had no effect. To evaluate the impact of a reduction in EAAT2 expression on exocytosis the two silencers were then transiently co-transfected in INS-1E cells with a plasmid encoding the human growth hormone (hGH). hGH release was used to selectively monitor exocytosis in the cells receiving the silencers, since hGH is targeted to β-cell secretory granules and co-released with insulin [22]. In cells transfected with the EAAT2-SilA we found that hormone secretion, evoked by high K^+^ (40 mM) and glucose (20 mM) concentrations (in the presence of the cAMP raising agents forskolin and IBMX: see Methods), was significantly reduced compared to that in mock (pSUPER) transfected cells or in cells receiving the inactive silencer (EAAT2-SilB). This effect was evident both during the first 10 min after raising the [K^+^] and [glucose] (first secretory phase) and during the following 35 min (sustained secretory phase) (Figure 6C). There was no effect of the EAAT2-SilA on basal insulin secretion (i.e. without the glucose/K^+^ stimulation regime, data not shown).

When the cells were stimulated with only a high glucose concentration (20 mM) the increase in secretion over basal secretion was significantly higher in the mock and EAAT2-SilB transfected cells (2.7- and 3.1-fold) than in EAAT2-SilA transfected cells (1.7-fold) (p<0.05, two-sample t-test). This was true also when only a high K^+^ concentration was used, for which the increase in secretion was significantly higher in the mock and EAAT2-SilB transfected cells (2.4-fold) than in cells treated with EAAT2-SilA (1.4-fold) (p<0.05). Furthermore, in INS-1E cells, in which exogenously transfected EAAT2 was expressed in secretory granules (Figure 6A), over-expression of EAAT2 caused a significant reduction in secretion (Figure 6D, an explanation of this effect is suggested below). Thus, EAAT2 activity contributes to regulation of secretion in β-cells, and either an increase or a decrease in its expression reduces secretion.

The low glutamate levels in insulin containing secretory granules, together with the observation that EAAT2 is not carrying glutamate into these granules, suggest that EAAT2 may optimize insulin secretion by generating an outward transport of glutamate, keeping the glutamate concentration low inside the secretory granules. To test this we expressed a glutamate transporter that carries glutamate into vesicles [2] in the INS-1E cells. All the cloned VGLUTs transport glutamate with similar substrate specificities and kinetics [2]. To ensure insertion of an excess of VGLUTs in the membranes of secretory granules we chose to use VGLUT2, which gave a high transfection yield, and is not expressed endogenously in β-cells. After transfection of INS-1E cells with c-myc tagged VGLUT2, the transporter was translocated to secretory granules as judged by its co-localization with insulin (Figure 6B). In such cells over-expressing a VGLUT, insulin secretion evoked by the high K^+^ and glucose concentrations was significantly reduced (Figure 6E). An explanation for this is suggested below.

### EAAT2 and VGLUT3 regulate the pH and membrane potential of the granules

To clarify the mechanism by which the VGLUT3 and EAAT2 glutamate transporters regulate the glutamate concentration in the granule and modulate exocytosis, we simulated the effect of including a VGLUT and EAAT2 in the secretory granule membrane on the pH and the membrane potential of the secretory granule (see Supplementary Text S1). With just the H^+^-ATPase in the granule membrane, the membrane becomes polarized to over +200 mV as soon as the ATPase is active, because there is no route for charge to leave the granule, which inhibits further ATPase activity (Figure 6G). Consequently, essentially no H^+^ or glutamate are accumulated (Figure 6H, I). Including a VGLUT in the membrane of the secretory granule provides a counter-charge movement (glutamate^−^ entering the granule; this may also occur via Cl^−^ channels: see Supplementary Text S1) which reduces the polarization of the granule membrane (Figure 6G) and thus allows maintained operation of the ATPase, leading to H^+^ accumulation via the ATPase (Figure 6H) and glutamate accumulation via the VGLUT (Figure 6I). Finally, inserting EAAT2 in addition to a VGLUT provides a mechanism for the release of accumulated glutamate (which would otherwise start to inhibit the VGLUT), and provides extra counter-charge movement out of the granule (because glutamate^−^ leaves the granule with 3Na^+^ and an H^+^, while 1 K^+^ enters the granule) leading to a less positive granule membrane potential (Figure 6G). Despite the efflux of H^+^ on EAAT2, the overall result is to increase the H^+^ concentration further (Figure 6H), while the glutamate level inside the granule is very much reduced (Figure 6I). Thus, including both glutamate transporters reduces the vesicle membrane potential and increases H^+^ accumulation. Consequently, glutamate may regulate exocytosis via two mechanisms. First, fluxing glutamate through the secretory granules via the two types of glutamate transporter promotes the acidification of the granules, which is known to be important both for the maturation of insulin [23] and for the exocytotic process [24]. Secondly, the concerted action of the two transporters limits the membrane potential reached across the secretory granule, which may be important for optimising exocytosis. An intact membrane potential across the granule, acting through trans-SNARE pairing mechanisms, is needed for exocytosis, possibly independent of the H^+^ accumulation produced by this potential [25]. Thus, the decrease of membrane potential that occurs when putting more EAAT2 and VGLUT into the secretory granules (Figure 6G) might explain why exocytosis is decreased by overexpression of these transporters (Figure 6D, E).

### The role of glutamate in modulating insulin release

The discussion above assumes that glutamate itself is not the initial trigger for the exocytosis of insulin-containing granules (we assume that the trigger is a glucose-evoked rise of ATP level closing ATP-gated K^+^ channels), but that the flux of glutamate through the secretory granules leads to the granules being more acidic and less positive in potential, and thus modulates the amount of insulin release occurring. Initial claims that glutamate potentiated secretion [9], [10], [26] were based on the observation that a rise in cytoplasmic glutamate concentration correlated with increased insulin release, but were disputed [27], [28] when it was found that insulin release did not always correlate with the total islet glutamate concentration. These discrepancies may be partly resolved by our data, which suggest that it is not so much the total islet, nor even cytoplasmic glutamate concentration that is the important regulatory factor, but the flux of glutamate through the secretory granules, which regulates the granule pH and membrane potential. Thus, we think that alteration of the glutamate flux through secretory granules is the mechanism by which altered glutamate levels modulate insulin release. Even though a simple relationship between cytoplasmic glutamate concentration and secretion is not to be expected, because the simulations depict that secretion is largely dependent on both the granule [H^+^] and its membrane potential, granule energetics could be affected by cytoplasmic glutamate levels (cytoplasmic glutamate must present to be transported through the granule). Consistent with this is recent data showing that knocking down a mitochondrial glutamate transporter (which will presumably alter the cytoplasmic glutamate level) also leads to a decrease of insulin secretion [30].

Another consequence of having EAAT2 in the granule membrane to maintain a low glutamate concentration in the granule could be that glutamate taken up into the granule is not lost into the extracellular space during granule exocytosis, but is recycled back to the cytoplasm where it may fuel the mitochondrial TCA cycle. Indeed, there is experimental evidence that β-cell secretory granules do not release a significant amount of glutamate [8]. In line with the present and previous in vitro results [9], [10], [26] we did not detect any changes in the basal (unstimulated) serum concentrations of insulin and glucose in EAAT2 KO mice (Figure S2). In summary, our data suggest that there is a glutamate cycle through insulin-containing secretory granules in β-cells, and that insulin secretion is modulated by the two types of glutamate transporter present in the secretory granules, which act in series to optimise the intragranular pH and the membrane potential across the secretory granules. Interestingly, the glutamate concentration within α- and β- cells seems to be regulated by the flux of the glutamate precursor glutamine between these cells [31].

A somewhat analogous role for VGLUT3 has recently been proposed for cholinergic synaptic vesicles in the striatum [29], where VGLUT3 mediated uptake of cytoplasmic glutamate amplifies cholinergic transmission by providing a compensatory anion influx to the vesicles, similar to what we propose for the insulin granules. Our data and simulations may therefore be important for understanding, not only the modulation of insulin secretion in the pancreas, but also the control of exocytosis of other membrane vacuole compartments more generally.

## Methods

### Ethics Statement

Animal experiments were carried out according to international legislations (European Communities Council Directive of 24 November 1986 (86/609/EEC)) and Norwegian (1996-01-15 no 23) and British (UK Animals (Scientific Procedures) Act 1986) regulations. Formal approval to conduct the experiments was obtained from the animal subjects review boards of our institutions. Every effort was made to minimize suffering and the number of animals used. The animals were maintained under standard colony conditions in a temperature- (23±1°C) and humidity- (40%) controlled animal room under a 12 h light/dark cycle (7:00–19:00), with ad libitum access to food and water.

### Primary antibodies

Polyclonal antibodies against L-glutamate and D-aspartate were raised in rabbits as originally described [32]. The no. 607 glutamate and the no. 482 D-aspartate antisera have been extensively characterized [16], [33]. The rabbit anti-73kDa antibodies to EAAT2 were raised against the EAAT2 protein isolated from rat brain [34]. A monoclonal EAAT2-antibody (9C4) binding to residues 518–525 (rat numbering) was produced in mouse [35], and a mouse monoclonal EAAT2 antibody was purchased from Novo Castra, UK. Anti-peptide antibodies against glutamate transporters were prepared as described [36]. Different EAAT2 antibodies were produced in rabbits against residues 12–26 (anti-B12) [36], residues 518–536 (anti-B518) [37], residues 493–517 (anti-B493) [37], or residues 563–573 (anti-B563). These, as well as the EAAT1 [34], EAAT3 [38] and EAAT4 [39] antibodies have been extensively charcterized. In addition, goat anti-EAAT2 antibodies (Santa Cruz Biotechnology, Santa Cruz, USA) were used in double labelling immunogold experiments.

Rabbit VGLUT1, VGLUT2 and VGLUT3 antibodies were gifts from R. H. Edwards (University of California, San Francisco, USA) [3], [17], [18]. Guinea pig VGLUT3 antibody was from Chemicon (Temecula, CA, USA).

A monoclonal mouse anti-glucagon antibody and polyclonal guinea pig anti-insulin antibodies were from Sigma (St. Louis, MO, USA), a monoclonal synaptophysin anitbody (mouse) from Boehringer Mannheim Biochemica (Mannheim, Germany), and a monoclonal mouse anti-insulin was from Zymed Laboratories, Inc. (San Francisco, USA). The anti-Myc mouse monoclonal antibody was purified from the culture medium of myeloma cells (clone 9E10) using an IgG affinity column.

### Tissue and cells

Adult Wistar rats were fed ad libitum and perfused [40] through the left cardiac ventricle with one of the following mixtures of fixatives in 0.1 M sodium phosphate buffer (pH 7.4) (NaPi): 2.5% glutaraldehyde and 1% formaldehyde (amino acid immunoperoxidase and immunogold cytochemistry), 4% formaldehyde and 0.1% glutaraldehyde (protein immunogold cytochemistry and immunofluorescence) or 4% formaldehyde (immunofluorescence). Wild type and KO mice [14] were fed ad libitum. After cervical dislocation (at P14), pancreatic and brain tissue from EAAT2 KO mice and wt littermates were immediately immersion fixed in a mixture of 4% formaldehyde and 0.1% glutaraldehyde. INS-1E and COS7 cells were cultured as previously described [41].

### Cellular uptake sites for D-aspartate

Pancreatic slices (0.5 mm thick) from adult Wistar rats were prepared by hand cutting, before they were transferred to vials with Krebs solution (pH 7.4 with 140 mM NaCl, 15 mM NaPi, 5 mM KCl, 5 mM glucose, 1.2 mM CaCl_2_, 1.2 mM MgSO_4_) that were continuously gassed with O_2_ at 30°C. The slices were preincubated for 30 minutes before incubation in the presence and absence of 100 µM D-aspartate for 20 minutes.

### D-asparate uptake in secretory granule

As D-aspartate is a substrate for EAAT2 [4], [5], [16], but not for the VGLUTs [3], [17], [18], we used D-aspartate as a tracer to explore whether secretory granules in β-cells show EAAT2 uptake activity. INS-1E cells were permeabilized with 1.5 IU/ml streptolysin-*O* and incubated with 0, 0.5, 0.3 and 1.0 mM D-aspartate in an intracellular medium containing 20 mM HEPES, pH 7.0, 140 mM KCl, 5 mM NaCl, 7 mM MgSO_4_, 5 mM Na_2_ATP, 10.2 mM EGTA and 0.1 mM CaCl_2_ for 7 min at 37°C.

Both the tissue slices and the INS-1E cells were fixed by either a mixture of 1% formaldehyde and 2.5% glutaraldehyde (for immunperoxidase and immunogold cytochemistry) or 4% formaldehyde and 0.5% glutaraldehyde (for immunfluorescence).

### Immunofluorescence and immunoperoxidase

After fixation, pancreas and brain specimens were cryostat sectioned (5–15 µm) after cryoprotection in 30% sucrose at 4°C overnight. The sections were processed with the antibodies according to an immunofluorescence or a streptavidin-biotin-peroxidase method as previously described [16], [31], [40]. The following antibody dilutions were used: rabbit anti-D-aspartate 482 (1∶1000), anti-glutamate 607 (1∶1000–1∶3000), and the anti-EAAT2 immunoglobulins anti-B12 (0.3–3 µg/ml), anti-B518 (1 µg/ml), anti-B493 (3 µg/ml), anti-B563 (0.3–1 µg/ml), mouse monoclonal EAAT2 antibody (1∶30), rabbit anti-EAAT1 (1–6 µg/ml), anti-EAAT3 (1–6 µg/ml), anti-EAAT4 (1–6 µg/ml), anti-VGLUT1 (1∶1000), anti-VGLUT2 (1∶2000), guinea-pig anti-VGLUT2 (1∶3000), anti-VGLUT3 (1∶300), guinea-pig anti-VGLUT3 (1∶1500), anti-insulin (1∶50–1∶100), anti-glucagon (1∶2000), mouse anti-c-Myc (1∶10). Primary antibodies were visualized with FITC-, Cy3- or Alexa (488 or 555)- fluorescent secondary antibodies.

In control immunofluorescence experiments in which the primary antibodies were omitted or substituted with preimmunesera, there was no significant staining of the tissue sections, but some experiments produced a diffuse fluorescence background that was subtracted from the labelling produced by the primary antibodies. In immunoperoxidase experiments, the glutamate and D-aspartate antisera were used with the addition of 0.2 mM complexes of glutaraldehyde/formaldehyde treated L-aspartate plus glutamine and L-aspartate, respectively. Addition of 0.3 mM complexes of L-glutamate and D-aspartate to the primary antibodies abolished the glutamate and D-aspartate labeling.

Sections were observed with a Zeiss Pascal laser scanning microscope or a Zeiss fluorescence microscope at wavelengths of 488 nm and 568 nm.

### Immunogold staining and quantitation

Tissue for electron microscopy was embedded in Lowicryl HM20 according to a freeze substitution protocol, and immunogold labelling was performed as described [40], [42]. Ultrathin sections were processed with the primary antibodies at the following dilutions: anti-D-aspartate (1∶300), anti-glutamate (1∶3000), anti-EAAT2 B12 (6–40 µg/ml) anti-EAAT2 73 kDa (5 µg/ml) and anti-VGLUT3 from guinea pig (1∶100). In single labelling experiments the primary antibodies were visualized with anti-rabbit or anti-guniea pig Ig specific secondary antibodies coupled to 15 nm gold particles (Europrobe, Lyon, France). In the double labelling experiments of KO and wt tissue, rabbit anti-glutamate (1∶3000) and goat anti-EAAT2 antibodies (2 µg/ml) were visualized with goat anti-rabbit (10 nm gold particles (BBI, UK)) and donkey anti-goat (15 nm gold particles (Aurion, The Netherlands)) secondary antibodies. In each amino acid experiment ultrathin test sections [43] were processed along with the islet sections, ascertaining the specificity of the labelling produced. When tested on ultrathin test sections containing known concentrations of glutamate and D-aspartate, both the glutamate and the D-aspartate antisera have been shown to produce a close to linear relation between the immunogold labelling densities and the concentrations of amino acids in the test sections [44], [45].

The sections were viewed in a Philips CM10 electron microscope and α- and β-cells were identified on morphological grounds [40], [46]. Immunogold particles signalling glutamate, EAAT2 and VGLUT3, and grid points for area determination [33], [40], [42] (see below) were recorded over secretory granules, SLMVs, plasma membranes, cytosol and mitochondria (background labelling). Particles and grid points were included in the SLMV when the particle centres or grid points were within a 30 nm distance from the outer border of the SLMV. This is a distance within which most of the immunogold particles are expected to occur [42], [47]. Similarily, EAAT2 and VGLUT3 gold particles with centres within 30 nm on either side of plasma membranes and membranes limiting secretory granules were recorded as belonging to these membranes (for details, see [40]). The EAAT2 and VGLUT3 values over tissue membranes were corrected for background labelling (measured over mitochondrial outer membranes: average 3.7 and 1.3 gold particles/µm^2^), while EAAT2 and VGLUT3 values over non-membrane compartments (cytosol, secretory granule cores) were corrected for background labelling measured over the mitochondrial matrix (average 4.9 and 2.7 gold particles/µm^2^). Similar quantifications were done for gold particles representing glutamate. The glutamate values were corrected for background labelling over empty resin (average 1.7 gold particles/µm^2^). The results were statistically evaluated by a non-parametric test (Mann-Whitney-U, two tails) (Statistica) and a paired-sample t-test (Excel).

### Western blotting

Pancreatic islets were acutely isolated as described [48]. Homogenates of isolated islets and COS-7 cells were separated by SDS-PAGE, blotted onto nitrocellulose, and proteins were immunodetected by primary antibodies, HRP-conjugated secondary antibodies and chemiluminescence reagents. COS-7 cell homogenates were also processed by secondary antibodies coupled to alkaline phosphatase (AP) and detected with AP-substrates.

### Cloning of rat EAAT2 and generation of EAAT2 silencers

The open reading frame of rat EAAT2 was cloned by PCR from an INS-1E cDNA library in pBK vector (Stratagene). The PCR reaction was performed using the forward primer 5′_CGCGGATCCATGGCATCAACCGAGGGT_3′ and the reverse primer 5′_GCTCTAGATTATTTTTCACGTTTCCA _3′. Both primers were designed according to the sequence of rat EAAT2 (NM_017215) [49]. For sequencing and expression experiments the PCR products were inserted in the BamHI and Xba I cloning sites of myc-pcDNA3. Sequence analysis of the inserts was performed by MWG Biotech Company (Germany). Mammalian expression vectors directing the synthesis of small interfering RNAs (siRNAs) targeted against EAAT2 were prepared according to the method of Brummelkamp [49]. Two cDNA fragments encoding a 19-nucleotide sequence derived from the target transcript and separated from its reverse 19-nucleotide complement by a short spacer were synthesized by MWG Biotech Company (Ebersberg, Germany), annealed and cloned in front of the H1-RNA promoter in the pSUPER vector^16^ nucleotides: 5′_GATCCCCCCGAGGGTGCCAACAATATTTCAAGAGAATATTGTTGGCACCC CGGTTTTTGGAAA_3′ and 5′_AGCTTTTCCAAAAACCGAGGGTGCCAACAATATTCTCTTGAAATATTGTT GCACCCTCGGGGG_3′ (SilA) and 5′_GATCCCCGATGCTCATCCTCCCTCTCTTCAAGAGAGAGAGGGAGGATGAGCATCTTTTTGGAAA _3′ and 5′_AGCTTTTCCAAAAAGATGCTCATCCTCCCTCTCTCTCTTGAAGAGAGGGAGGATGAGCATCGGG_3′ (SilB). To test for the silencing activity of SilA and SilB, each plasmid was transiently co-transfected with the plasmid encoding rat myc-tagged EAAT2 in COS 7 cells. The expression of EAAT2 was assessed two days later by Western blotting.

### Secretion assay

INS-1E cells were transiently cotransfected with a plasmid encoding human growth hormone (hGH) and with plasmids encoding the siRNAs or EAAT2-c-Myc or VGLUT2-green fluorescent protein (GFP) constructs. Three days later, the cells were preincubated for 30 min in 20 mM HEPES, pH 7.4, 128 mM NaCl, 5 mM KCl, 1 mM MgCl_2_, 2.7 mM CaCl_2_ and 2 mM glucose. The cells were then incubated for 45 min at 37°C either in the same buffer or stimulated with the 20 mM HEPES buffer containing 40 mM KCl, 20 mM glucose, 1 µM forskolin, and 1 mM 3-isobutyl-L-methylxantine (IBMX). In some experiments the cells were stimulated with either 20 mM glucose or 40 mM K^+^ alone. Exocytosis from transfected cells was determined by measuring by ELISA the amount of hGH (Roche, Rotkreuz, Switzerland) released into the medium during the incubation period, and expressed as an increase over basal (exocytosis = (stimulated−basal)/basal). The results were statistically evaluated by a two-sample t-test.

At the time of stimulation some cells were fixed with 4% formaldehyde and processed for confocal immunofluorescent microscopy with c-Myc and insulin antibodies.

Blood samples from EAAT2 KO mice and littermates (n = 8 of each +/+ and −/−) were taken from the aorta after cervical dislocation and serum was prepared for ELISA measurements. Insulin was analysed using the Mouse Ultrasensitive Insulin ELISA kit (Mercodia, Uppsala, Sweden). Serum samples for each animal were analysed in triplicate and absorbance read at 450 nm in a Photometer for microtitration plate. Blood glucose concentrations from each sample were analysed by Accu-Chek compact (Roche Diagnostics GmbH, Mannheim, Germany).

### RT-PCR

PCR was conducted on RNA from isolated islets of Langerhans and brain tissue using Qiagen OneStep RT-PCR kit (35 3-step cycles). Primer control and -RT controls were included. Primers used were EAAT2 P1F (GAAAAAACCCATTCTCCTTTTT) and EAAT2 P1R (CCGACTGGGAGGACGAATC). Primers were from Oligold Eurogentec. 20 ng RNA template of each tissue was used per reaction. Electrophoresis was conducted on 2% agarose gels, samples diluted with Fermentas 6× Orange buffer.

### Simulation of granule energetics

Details of these simulations are given in the Supplementary Material.

## Supporting Information

Figure S1 The figure shows the subcellular distribution of VGLUT3 and EAAT2 in INS1-E cells. After elimination of nuclei and cell debris the postnuclear supernatants from INS1-E cells were separated on a sucrose density gradient (0.45–2 M). (A) The insulin content was measured by ELISA in each fraction of the gradient. Dense-core insulin-containing secretory granules are recovered in the fractions corresponding to 1.3–1.6 M sucrose. (B) The fractions were analysed by Western blotting using antibodies against VGLUT3, EAAT2 and synaptophysin. In contrast to insulin, synaptophysin, a marker of synaptic-like microvesicles, is recovered at 0.9–1.3 M sucrose. EAAT2 was located in the insulin containing fraction, suggesting that it is present in insulin granules. VGLUT3 was located both in the insulin and the synaptophysin containing fractions, suggesting that it is present in secretory granules and SLMVs. Method: Subcellular fractionation of approximately 10^8^ INS-1E cells was performed as described (Ref 1 Supplementary Text S1). Briefly, a postnuclear supernatant obtained after disruption of the cells by sonication, was loaded on a continuous sucrose density gradient (8 ml; 0.45–2 M sucrose). After centrifugation for 18 h at 110,000× *g*, 16 fractions of 0.5 ml were collected from the top of the tube. The concentration of sucrose in the fractions was determined by measuring the refractive index of the solution. The amount of insulin present in the fractions was measured by ELISA (Mercodia). The distribution throughout the gradient of EAAT2, VGLUT3 and synaptophysin was measured by Western blotting. The bound antibody was visualized using an HRP-conjugated goat anti mouse (synaptophysin) and rabbit anti-goat (EEAT2, VGLUT3) followed by chemiluminescence reagents.
(TIF)

Figure S2 To examine whether EAAT2 affects insulin secretion in intact animals, we analysed insulin serum concentrations in the EAAT2 KO mice (KO), in which the transporter is absent from the β-cell SGs (see Fig. 4), and compared these values to results obtained in wild type mice (wt). The values are the mean serum concentration of glucose and insulin ± SD in 8 wt and 8 KO animals. We could not detect any significant difference in insulin or glucose concentrations between mice lacking and expressing EAAT2. As the serum glucose concentrations in the EAAT2 KO mice is low (4.9±0.7 mM) the hormone values reflect basal secretion, which has been shown not to be significantly affected by glutamate (see text). This is also in accordance with our in vitro data showing that silencing of EAAT2 is not important for regulating basal insulin secretion. We are not in a position to keep the EAAT2 knock out animals for doing in vivo animal research. The EAAT2 KO mice get epileptic after 2 weeks of age and, according to the animal welfare regulations that we presently have to abide by, they must be killed before the age of 2 weeks. This precludes further detailed analysis, such as insulin measurements after glucose tolerance tests, which would be extremely demanding to do on mice younger than 14 days of age.
(TIF)

Text S1 (DOC)
