# Supplementary material for: A Role for Glutamate Transporters in the Regulation of Insulin Secretion
Source: PLoS One. 2011 Aug 11;6(8):e22960. doi: 10.1371/journal.pone.0022960 (PMC3154915; doi:10.1371/journal.pone.0022960)
Supplement: Text S1 — (DOC) [file pone.0022960.s003.doc]

**SUPPLEMENTARY TEXT S1**

**Simulation of granule energetics**

Simulations of granule cell pH and membrane potential were carried out using MathCad (MathSoft), by simultaneously solving differential equations describing the granule membrane potential and intracellular ion and glutamate concentrations. We initially assumed that the H^+^-ATPase can pump protons into a granule of diameter 305nm [2] at a maximum rate which would raise the free [H^+^] in the vesicle at 0.1μM/sec (sufficient to produce a pH of 5 in 100 sec if no H^+^ leave the granule), assuming that only 1 in 4000 H^+^ entering remains unbuffered (corresponding to a buffering power of ~40mM/pH unit [3] around pH 6), i.e. a proton current of I_max_=5.7.10^-16^ amps (or 3582 protons/sec). For consistency with the thermodynamic constraint that the ATPase can only pump protons against the transmembrane [H^+^] and voltage gradient if the energy available from ATP is greater than the energy needed to move an assumed 2 H^+^/ATP across the membrane, this ATPase-mediated proton current was multiplied by a factor

(E_ATP_ – 2RT.ln([H^+^]_i_/[H^+^]_o_) – 2FV) / E_ATP_,

where E_ATP_ is the energy provided per ATP (50kJ/mole), and the subsequent terms are the energy needed to accumulate 2 H^+^ against the [H^+^] and voltage gradient across the granule membrane (R is the gas constant, T is temperature, F is the Faraday, [H^+^]_i_ is the free proton concentration in the vesicle, [H^+^]_o_ is the cytoplasmic [H^+^], and V is the potential inside the vesicle relative to the cytoplasm: the exact expression assumed to define the thermodynamic constraint is not critical for the results obtained). As [H^+^]_i_ and V increase, the two subsequent terms become significant compared to E_ATP_ and proton pumping is reduced. A specific membrane capacitance of 1μF/cm^2^ was assumed [4], implying a granule capacitance of 2.9x10^-15^F, for the equation relating rate of change of membrane potential to transmembrane ionic current. Immediately after exocytosis, when the granule interior is in contact with the extracellular space, the granule membrane potential was set to the assumed cell membrane potential (+70mV for a cell membrane potential of -70mV since we define the granule membrane potential as that voltage inside the granule minus the voltage in the cytoplasm; this value is not critical for the results) and its contents were assumed to be similar to the extracellular space (140mM Na^+^, 3.5mM K^+^, and 100μM residual glutamate: the exact value assumed for this low level of residual [glutamate] did not critically affect the glutamate concentration reached inside the granule during ATPase activity). The ATPase was then allowed to run for 200 sec, resulting in accumulation of H^+^ and generation of a membrane potential. With only the ATPase in the membrane the membrane potential rapidly reached over 200mV and this inhibited the ATPase (Fig. 6G).

A VGLUT was then added, represented as a membrane conductance through which glutamate moves and generates a current given by: g_Glu_.(V-V_glu_), where V_glu_ is the Nernst potential for glutamate across the granule membrane and g_Glu_ was set to 4.8x10^-16^ Siemens (i.e. sufficient to significantly affect the granule membrane potential produced by the ATPase). The cytoplasmic [glutamate] was assumed to be 5mM. Similarly in some simulations a conductance to H^+^, Cl^-^ or K^+^ was added, to provide extra charge compensation for the current generated by the ATPase (for example charge compensation may occur via CLC-3 Cl^-^ channels [5] or through the VGLUT itself [6]): provided the currents mediated by H^+^, Cl^-^ and K^+^ were not large compared with those mediated by glutamate this did not alter the effects of adding the VGLUT or EAAT2 (results not shown).

Finally EAAT2 was added. Its glutamate flux was assumed to be given by a constant defining the maximal unidirectional flux (described below) multiplied by the difference of two terms (representing efflux and influx) with a dependence on ion concentrations on each side of the membrane as follows (in accordance with the fact that each glutamate anion is co-transported with 3 Na^+^ and 1 H^+^, while 1 K^+^ is counter-transported [7])

where the subscripts i and o represent inside and outside (in the cytoplasm) the granule, glu, Na, K and H represent concentrations of these species, and the cytoplasmic [glu^-^], [Na^+^], [K^+^] and [H^+^] were set to 5mM, 20mM, 140mM and 10^-7^M respectively. The EC_50_ values for activation of transport were set to K_glu_=20μM, K_Na_=20mM, K_H_=10^-7^M, and K_K_=20mM respectively [8, 9, 10], and the efflux and influx pathways were arbitrarily assumed to have the same voltage dependence. In the simulations, setting the influx (second) term in the equation to zero made little difference because influx never occurs via EAAT2.

The equation above is not consistent with the thermodynamic equation for the glutamate concentration ratio produced across the granule at equilibrium (a detailed model of the transporter would be needed to produce an equation that is both consistent with thermodynamics and provides a realistic saturating dependence of transport on substrate concentrations as in the equation above), but further simulations consistent with the thermodynamic equilibrium condition, in which glutamate flux was represented as proportional to a constant conductance multiplied by the thermodynamic driving force for the transporter (the difference between the membrane potential and the reversal potential for the transporter, i.e. (3V_Na_+V_H_-V_K_-V_glu_)/2, see [7, 11]), gave results similar to those shown in Fig. 6 for the effects of including the VGLUT and EAAT2.

The constant describing the maximal unidirectional flux which multiplies the equation above was assumed to produce a current (generated by the net transport of 2 elementary charges) of 5x10^-18^ amps at 0mV, i.e. chosen to be sufficient to affect the accumulated level of glutamate and [H^+^]. Simulating the (13mM) rise in granule [K^+^] produced by EAAT2 had essentially no effect on the accumulation of H^+^ and glutamate produced; the corresponding fall of granule [Na^+^] (from 140mM to 101mM) associated with EAAT2 exporting glutamate was ignored as the Na^+^ site is closer to saturation.

**Supplementary References**

1. Regazzi R, Ravazzola M, Iezzi M, Lang J, Zahraoui A, Andereggen E, Morel P, Takai Y, Wollheim CB (1996) Expression, localization and functional role of small GTPases of the Rab3 family in insulin-secreting cells. J Cell Sci 109: 2265-2273.

2. Gopel S, Zhang Q, Eliasson L, Ma XS, Galvanovskis J, Kanno T, Salehi A, Rorsman P (2004) Capacitance measurements of exocytosis in mouse pancreatic alpha-, beta- and delta-cells within intact islets of Langerhans. J Physiol 556: 711-726

3. Grabe M, Oster G (2001) Regulation of organelle acidity. J Gen Physiol 117: 329-344

4. [Ammälä C](http://www.ncbi.nlm.nih.gov/sites/entrez?Db=pubmed&Cmd=Search&Term="Ammälä C"%5BAuthor%5D&itool=EntrezSystem2.PEntrez.Pubmed.Pubmed_ResultsPanel.Pubmed_DiscoveryPanel.Pubmed_RVAbstractPlus), [Eliasson L](http://www.ncbi.nlm.nih.gov/sites/entrez?Db=pubmed&Cmd=Search&Term="Eliasson L"%5BAuthor%5D&itool=EntrezSystem2.PEntrez.Pubmed.Pubmed_ResultsPanel.Pubmed_DiscoveryPanel.Pubmed_RVAbstractPlus), [Bokvist K](http://www.ncbi.nlm.nih.gov/sites/entrez?Db=pubmed&Cmd=Search&Term="Bokvist K"%5BAuthor%5D&itool=EntrezSystem2.PEntrez.Pubmed.Pubmed_ResultsPanel.Pubmed_DiscoveryPanel.Pubmed_RVAbstractPlus), [Larsson O](http://www.ncbi.nlm.nih.gov/sites/entrez?Db=pubmed&Cmd=Search&Term="Larsson O"%5BAuthor%5D&itool=EntrezSystem2.PEntrez.Pubmed.Pubmed_ResultsPanel.Pubmed_DiscoveryPanel.Pubmed_RVAbstractPlus), [Ashcroft FM](http://www.ncbi.nlm.nih.gov/sites/entrez?Db=pubmed&Cmd=Search&Term="Ashcroft FM"%5BAuthor%5D&itool=EntrezSystem2.PEntrez.Pubmed.Pubmed_ResultsPanel.Pubmed_DiscoveryPanel.Pubmed_RVAbstractPlus), [Rorsman P](http://www.ncbi.nlm.nih.gov/sites/entrez?Db=pubmed&Cmd=Search&Term="Rorsman P"%5BAuthor%5D&itool=EntrezSystem2.PEntrez.Pubmed.Pubmed_ResultsPanel.Pubmed_DiscoveryPanel.Pubmed_RVAbstractPlus) (1993) Exocytosis elicited by action potentials and voltage-clamp calcium currents in individual mouse pancreatic B-cells. J Physiol 472: 665-688

5. Barg SP, Eliasson L, Nelson DJ, Obermuller S, Rorsman P, Thevenod F,

Renstrom E (2001) Priming of insulin granules for exocytosis by granular Cl^-^ uptake and acidification. J Cell Sci 114: 2145-2154

6. Schenck S, Wojcik SM, Brose N, Takamori S (2009) A chloride conductance in VGLUT1 underlies maximal glutamate loading into synaptic vesicles. Nature Neurosci 12: 156-162

7. Levy L, Warr O, Attwell D (1998) Stoichiometry of the glial glutamate transporter GLT-1 expressed inducibly in a Chinese hamster ovary cell line selected for low endogenous Na+-dependent glutamate uptake. J Neurosci 18: 9620-9628

8. Brew H, Attwell D (1987) Electrogenic glutamate uptake is a major current carrier in the membrane of axolotl retinal glial cells. Nature 327: 707-709

9. Barbour B, Brew H, Attwell D (1988) Electrogenic glutamate uptake in glial cells is activated by intracellular potassium. Nature 335: 433-435

10. Billups B, Attwell D (1996) Modulation of non-vesicular glutamate release by pH. Nature 379: 171-174

11. Zerangue N, Kavanaugh MP (1996) Flux coupling in a neuronal glutamate transporter. Nature 383: 634-637
